# Melatonin Enhances Maize Germination, Growth, and Salt Tolerance by Regulating Reactive Oxygen Species Accumulation and Antioxidant Systems

**DOI:** 10.3390/plants14020296

**Published:** 2025-01-20

**Authors:** Wei-Qing Li, Jia-Yu Li, Shao-Jie Bi, Jia-Yue Jin, Zhong-Ling Fan, Zi-Lin Shang, Yi-Fei Zhang, Yan-Jie Wang

**Affiliations:** 1 College of Agriculture, Heilongjiang Bayi Agricultural University, Daqing 163319, China; lwq_ljy@163.com (W.-Q.L.); byndljy@163.com (J.-Y.L.); byndzyf@163.com (Y.-F.Z.); 2 College of Life Science and Biotechnology, Heilongjiang Bayi Agricultural University, Daqing 163319, China; bishaojie1990@163.com (S.-J.B.); jinjiayue2015@163.com (J.-Y.J.); fan00232024@163.com (Z.-L.F.); 13154548513@163.com (Z.-L.S.); 3 Heilongjiang Provincial Key Laboratory of Environmental Microbiology and Recycling of Argo-Waste in Cold Region, Daqing 163319, China

**Keywords:** melatonin, salt stress, maize (*Zea mays* L.), reactive oxygen species, antioxidant system

## Abstract

Melatonin (MT) is a crucial hormone that controls and positively regulates plant growth under abiotic stress, but the biochemical and physiological processes of the combination of melatonin seed initiation and exogenous spray treatments and their effects on maize germination and seedling salt tolerance are not well understood. Consequently, in this research, we utilized the maize cultivars Zhengdan 958 (ZD958) and Demeiya 1 (DMY1), which are extensively marketed in northeastern China’s high-latitude cold regions, to reveal the modulating effects of melatonin on maize salinity tolerance by determining the impacts of varying concentrations of melatonin on maize seedling growth characteristics, osmoregulation, antioxidant systems, and gene expression. The findings revealed that salt stress (100 mM NaCl) significantly inhibited maize seed germination and seedling development, which resulted in significant increases in the H_2_O_2_ and O_2_^−^ content and decreases in the antioxidant enzyme activity and photosynthetic pigment content in maize seedlings. However, exogenous melatonin considerably reduced the development inhibition caused by salt stress in maize seedlings. Moreover, exogenous melatonin alleviated NaCl-induced membrane damage and oxidative stress, and reduced Na^+^ content and excessively large quantities of reactive oxygen species (ROS). In addition, exogenous melatonin increased antioxidant enzyme activity and the expression of the antioxidant enzyme genes *ZmSOD4*, *ZmCAT2*, and *ZmAPX2*. This study demonstrates the potential role of combined melatonin seed initiation and foliar spray treatments in mitigating the detrimental effects of salt stress on maize growth, giving a theoretical foundation to future research on the possible advantages of exogenous regulating chemicals in attaining sustainable production in salt-alkaline soils.

## 1. Introduction

An increased soil salinity is a significant environmental element that affects agricultural production and is extremely harmful to crop growth, seriously affecting the sustainable development of agriculture and future food safety [1]. At the global level, about 45 million liters of agricultural land are affected by salinity, with an impact area of 0.2–0.5 million liters per year [2]. Over 20 per cent of land used for agriculture has been impacted by increased salinity to date [3,4]. Salinity-affected soils contain high levels of soluble salts, especially in soil conditions characterized by NaCl, which reduce plant growth capacity, photosynthetic efficiency, and nutrient uptake by inducing osmotic stress, ionic imbalances, and alterations in plant metabolic processes, and in severe cases, lead to plant death [5].

Salt stress causes osmotic stress, ionic imbalance, and oxidative damage to plants, reduces the ability of plants to absorb water and nutrients from the soil, inhibits seed germination, growth, and development, leads to the stunting of plant growth and the wilting of leaves, and, finally, leads to a decrease in crop yield [6,7,8,9,10]. The ionic toxicity induced by high concentrations of Na^+^ and Cl^−^ damages the balance of cells, increases the extent of membrane lipid damage, and inhibits photosynthesis [11,12], also causing physiological drought by decreasing the soil solution’s osmotic potential [13]. Salt stress prevents plants from taking up water from the soil, leading to a decrease in cellular water, which affects cell expansion and leads to a high levels of reactive oxygen species (ROS) accumulation, which affects normal plant growth [14,15]. However, in order to cope with salt stress, a complex system has evolved in plants, which includes the control of osmotic potential, ionic balance, endogenous hormone levels, and antioxidant enzyme activities [16]. Through the salt-sensitive (SOS) signaling system, which is activated by Ca^2+^ in the cytoplasm, plants regulate ion uptake and transport. This mechanism can maintain Na^+^/K^+^ in the cell by exporting excess Na^+^ [17,18]. In addition, by scavenging excess accumulated ROS, the ROS scavenging system, which is mediated by antioxidant enzymes such as ascorbate peroxidase (APX), superoxide dismutase (SOD), peroxidase (POD), and catalase (CAT), can lower the degree of cellular damage. This is an essential system for reducing oxidative harm to plants [19].

Melatonin (N-acetyl-5-methoxytryptamine) is a multi-signaling pathway phytohormone that plays an irreplaceable role in the life processes of plants and animals, and it is involved in seed germination, lateral root formation, photosynthesis, senescence, and responses to various environmental stresses, including immune enhancement, antioxidant activity regulation, and seasonal reproduction [20,21,22,23,24]. The most cyclical function of melatonin is to protect plants from biotic and abiotic stresses, which can enhance seed germination, leaf growth, and the ability to increase antioxidant capacity and stress tolerance in plants under abiotic stresses [25,26]. Melatonin has been shown to be a key regulator of plant growth and development and various environmental stresses [27,28], in addition to acting as a signaling molecule involved in defense responses to abiotic stresses such as salinity, alkalinity, low temperature, and drought [29,30,31]. Melatonin has been reported to maintain ROS homeostasis in plants under abiotic stress by reducing chlorophyll degradation, delaying leaf senescence, and increasing antioxidant capacity [16,32]. Exogenous melatonin can enhance alfalfa growth under salt stress by scavenging excessive H_2_O_2_ accumulation, enhancing antioxidant enzyme activities, and up-regulating the expression of antioxidant-enzyme-synthesizing genes [25]. One of the most important crop species in the world is maize (*Zea mays* L.), a major source of feed and industrial feedstuffs [33]. The increasing global population and living standards, along with the increasing demand for maize products, make it essential to ensure maize production [34]. It has been reported that maize is sensitive to severe reductions in growth and yield caused by high salt levels in the soil [35]. In order to deal with the problem of salt stress, many studies have focused on the use of exogenous regulatory substances to improve crop tolerance [36]. Yan et al. found that the exogenous spraying of melatonin significantly reduced the inhibitory effect of salt stress on the wheat development of seedlings [37]. The exogenous spraying of melatonin mitigates the detrimental action of salt stress through reducing the oxidative damage caused by salt stress, thereby promoting the growth of seedlings of plants such as alfalfa [25] and maize [38]. In addition, seed priming with melatonin alleviated the adverse consequences of PEG-6000-induced drought stress on black wheat seedlings and resulted in a significantly greater accumulation of biomass and antioxidant activity in the seedlings [39]. Considering that the response of maize to salinity, as well as melatonin, may vary depending on the stage of growth, in this study, we used the main cultivars Zhengdan 958 (ZD958) and Demeiya 1 (DMY1), which are widely promoted in the high-latitude cold regions of northeastern China and have salinity and drought resistance, in the present research to better understand the function of melatonin in regulating the germination and the growth of the seedlings of both of the above-mentioned maize varieties under salt stress. The theoretical underpinnings and technical references for the future use of melatonin as an exogenous regulating agent to enhance the germination properties of the two maize varieties mentioned above, as well as in high-salinity environments, will be provided by this study.

## 2. Results

### 2.1. Inhibition of Maize Seed Germination by NaCl Reversed by Melatonin Priming

**Figure 1:**
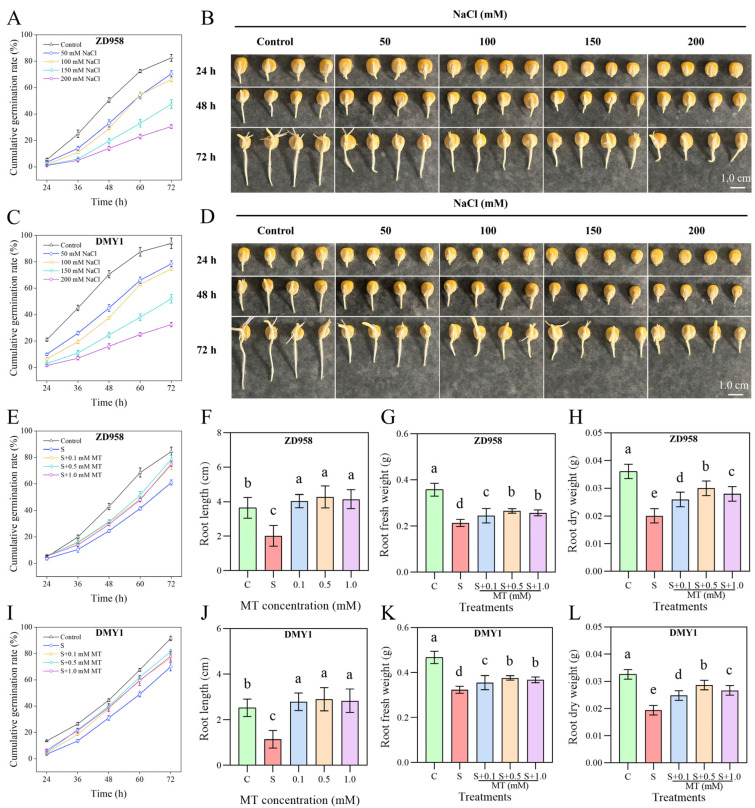
Maize seed germination under different concentrations of NaCl (salt stress) and melatonin (MT) priming treatments. (**A**,**C**) ZD958 and DMY1 cumulative germination rates per 12 h for a total of 72 h under 0, 50, 100, 150, and 200 mM NaCl treatments; (**B**,**D**) changes in germination characteristics of maize seeds at 24, 48, and 72 h under 0, 50, 100, 150, and 200 mM NaCl treatments by ZD958 and DMY1; (**E**,**I**) impacts of varying quantities of MT initiation treatments (0.1, 0.5, and 1.0 mM) on the cumulative germination rate of ZD958 and DMY1 maize seeds per 12 h for a period of 72 h under stress from salt; (**F**,**J**) the implications of varying concentrations of MT primed treatments (0.1, 0.5, and 1.0 mM) on radicle length of ZD958 and DMY1 maize seeds under salt stress; (**G**,**K**) the implications of varying concentrations of MT priming treatments (0.1, 0.5, and 1.0 mM) on the fresh weight of radicles of ZD958 and DMY1 maize seeds under salt stress; and (**H**,**L**) the implications of varying concentrations of MT-priming treatments (0.1, 0.5, and 1.0 mM) on dry weight of radicles of ZD958 and DMY1 maize seeds under salt stress. The data are presented as means ± SE (n = 4); significant differences between treatments are indicated by different letters (*p* < 0.05), scale bar: 1.0 cm.

Using sterile water (control) and four different concentrations of NaCl solutions, the effects of NaCl on the pattern of maize seed germination and radicle elongation were investigated. In control condition, the rates of germination of ZD958 and DMY1 at 24, 36, 48, 60, and 72 h were 5.50%, 25.00%, 50.50%, 72.50%, and 82.50% and 21.00%, 45.00%, 70.50%, 87.50%, and 94.00%, respectively (Figure 1A,C). The seed germination rates of the two varieties incubated up to 36–72 h under different concentrations of NaCl treatment conditions showed significant variations from the control treatment, which decreased under the state of added NaCl treatment, and the inhibition of the seed germination process was gradually strengthened as the concentration of NaCl decreased. After a 72 h incubation period, the germination rates of ZD958 and DMY1 were 70.50%, 66.00%, 47.50%, and 30.50% and 78.50%, 74.50%, 52.00%, and 32.50% when treated with 50, 100, 150, and 200 mM NaCl solution, respectively. At 72 h, the germination rate of ZD958 under the condition of 150 mM NaCl solution was 47.50%, which did not reach 50.00%. Therefore, in this study, we chose to treat with 100 mM NaCl solution to further investigate the relationship between the regulatory actions of NaCl and MT in maize seed germination and seedling growth.

In order to evaluate how MT affects maize seed germination and seedling development, germination tests were conducted on maize seeds initiated with different concentrations of MT (0, 0.1, 0.5, and 1.0 mM) under salt stress in the control and 100 mM NaCl conditions. MT was found to differentially increase the pace at which the maize seedlings germinated when exposed to salt stress at specific time points. At 72 h, the 0.1, 0.5, and 1.0 mM MT priming treatments raised the rates of germination of ZD958 and DMY1 by 21. 31%, 28.69%, and 22.95% and by 9.29%, 16.43%, and 11.43%, respectively, compared with the S treatment (Figure 1E,I). In addition, MT priming also promoted the elongation of maize radicles, and MT priming treatments at 0.1, 0.5, and 1.0 mM lengthened the ZD958 and DMY1 radicles by 100.04%, 111.78%, and 105.35% and by 144.39%, 153.96%, and 148.02%, respectively (Figure 1F,J). Compared to maize radicles grown under salt stress, the fresh weight of radicles primed by varying concentrations of MT was greater, and the dry weight was, likewise, marginally higher. These findings show that melatonin, of which 0.5 mM MT was the most effective, may lessen the suppression of maize seed germination by salt stress.

### 2.2. Melatonin Promotes the Growth of Maize Seedlings Under Salt Stress

**Figure 2:**
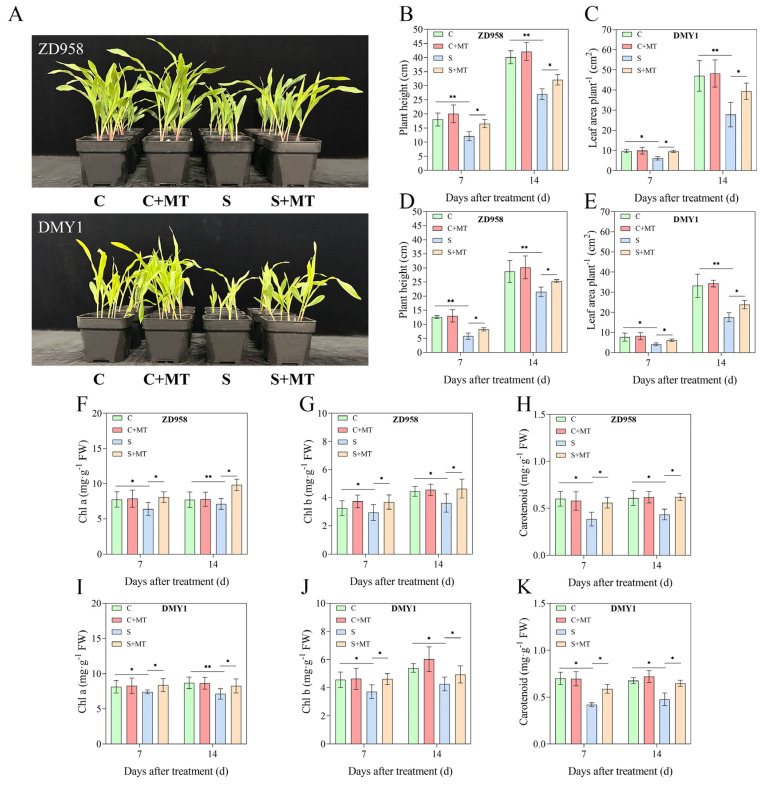
Effects of several treatments (C, C+MT, S, and S+MT) on maize seedling growth and photosynthetic hormones. (**A**) Changes in morphology of ZD958 and DMY1 seedlings after 7 d of exogenous melatonin spraying under control and salt stress; (**B**,**D**) changes in plant height of ZD958 and DMY1 after 7 and 14 d of melatonin spraying; (**C**,**E**) changes in leaf area of ZD958 and DMY1 after 7 and 14 d of melatonin spraying; (**F**,**I**) changes in leaf chlorophyll a content of ZD958 and DMY1 after 7 and 14 d of melatonin spraying; (**G**,**J**) changes in leaf chlorophyll b content of ZD958 and DMY1 after 7 and 14 d of melatonin spraying; and (**H**,**K**) changes in carotenoid content in leaves of ZD958 and DMY1 after 7 and 14 d of melatonin spraying. Asterisks (*) denote significant differences between treatments, and the data are presented as means ± SE (n = 4) (* *p* < 0.05, ** *p* < 0.01).

There were significant differences in the growth phenotypes of the maize seedlings after 7 d of growth under the S treatment conditions (Figure 2A). Compared with the C treatment, the maize seedling plant height and leaf area were significantly reduced (*p* < 0.05) after 7 and 14 d of incubation in the S treatment, and the plant height decreased by 28.55% and 29.94% and 60.42% and 22.86% in the ZD958 and DMY1 seedlings under salt stress, respectively (Figure 2B,D), while the leaf area decreased in the ZD958 and DMY1 seedlings under salt stress by 36.83% and 40.91% and 46.21% and 47.17%, respectively, indicating that DMY1 appeared to be more sensitive to salt stress compared to ZD958. However, the exogenous spraying of melatonin significantly promoted the growth of the seedlings of the two maize varieties under salt stress, but did not significantly promote the growth of maize seedlings under normal conditions. The plant height of the ZD958 and DMY1 seedlings increased by 12.92% and 17.76%, and the leaf area increased by 41.68% and 35.96%, respectively, after 14 d of S+MT treatment compared with the S treatment. In addition, the S treatment significantly reduced the chlorophyll a, chlorophyll b, and carotenoid contents of the maize seedlings compared to the C treatment (Figure 2F–K). When incubated for 7 and 14 d, chlorophyll a, chlorophyll b, and carotenoids were reduced by 17.48%, 9.40%, and 36.03% and 7.80%, 18.66%, and 28.81%, respectively, in the ZD958 seedlings under the S treatment conditions, and 6.04%, 18.52%, and 39.88% and 15.52%, 14.43%, and 29.42%, respectively, in the DMY1 seedlings, as compared with the C treatment. However, the S+MT treatment significantly increased the chlorophyll a, chlorophyll b, and carotenoid contents compared to the S treatment, both when incubated up to 7 d and 14 d. The chlorophyll a, chlorophyll b, and carotenoid contents of the ZD958 seedlings increased by 26.38%, 25.31%, and 45.48% and 38.31%, 28.30%, and 43.35%, and those of DMY1 seedlings by 13.01%, 24.00%, and 39.97% and 15.71%, 15.85%, and 35.68%, respectively, with incubation times up to 7 and 14 d.

### 2.3. Melatonin Inhibits the Production of Oxidative Stress Compounds

**Figure 3:**
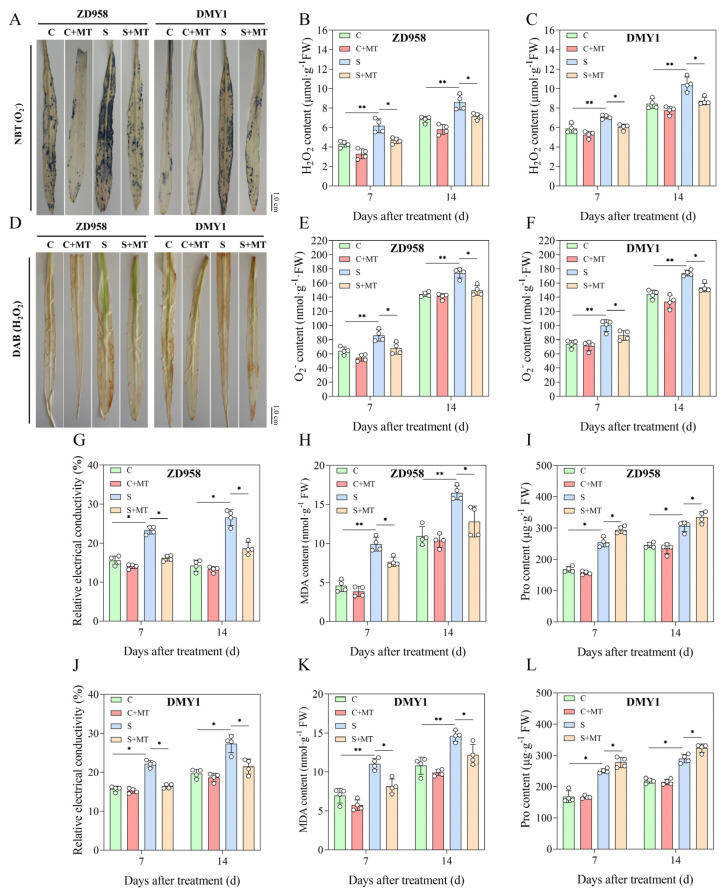
Effect of melatonin exogenous spraying on ROS levels and osmoregulatory compounds in salt-stressed maize seedlings. (**A**,**D**) Histochemical staining of leaves of ZD958 and DMY1 maize seedlings with blue-purple spots at the O_2_^−^ level and dark brown spots at the H_2_O_2_ level; (**B**,**C**) quantification of H_2_O_2_ in leaves of ZD958 and DMY1 maize seedlings; (**E**,**F**) quantification of O_2_^−^ in leaves of ZD958 and DMY1 maize seedlings; (**G**,**J**) effects of exogenously applied melatonin on ZD958 and DMY1 maize seedlings leaves relative conductivity under salt stress; (**H**,**K**) effects of melatonin exogenous spraying on MDA levels in ZD958 and DMY1 maize seedling leaves during salt stress; and (**I**,**L**) effects of melatonin exogenous spraying on Pro content in ZD958 and DMY1 maize seedlings’ leaves during salt stress. Asterisks (*) denote significant differences between treatments, and the data are presented as means ± SE (n = 4) (* *p* < 0.05, ** *p* < 0.01), scale bar: 1.0 cm.

Large concentrations of ROS in plants can have a negative impact on their growth and development under unfavorable environments [40]. In the present study, H_2_O_2_ and O_2_^−^ accumulation gradually increased in both maize varieties under each treatment condition as the treatment time increased. Compared with the C treatment, salt stress (S) resulted in a significantly higher H_2_O_2_ and O_2_^−^ in the maize seedlings (Figure 3B–F), in which the H_2_O_2_ and O_2_^−^ contents of ZD958 increased by 43.58% and 33.84%, respectively, and those of DMY1 increased by 20.27% and 35.69%, respectively, after 7 d of S treatment, which indicated that salt stress caused ZD958 to accumulate more ROS during growth and produce a stronger growth inhibition. Compared with the S treatment, the S+MT treatment, on the other hand, significantly reduced the H_2_O_2_ and O_2_^−^ content in both varieties, with ZD958 seeing reductions in its H_2_O_2_ and O_2_^−^ content by 16.47% and 14.14%, respectively, and DMY1 by 16.56% and 11.77%, respectively, after 14 d of treatment. In addition, blue-purple and dark brown precipitates gradually increased in the leaves of the maize seedlings under the S treatment conditions, but blue-purple and dark brown precipitates gradually decreased in the leaves after the S+MT treatment (Figure 3A,D), which indicates that melatonin reduced the H_2_O_2_ and O_2_^−^ contents of the leaves, which is consistent with the results of the H_2_O_2_ and O_2_^−^ quantification and indicates that the exogenous spraying of melatonin was effective in decreasing salt accumulation due to salt stress.

Furthermore, we determined the conductivity, MDA, and Pro content of the maize seedlings after different treatments. In the leaves of the maize seedlings under the control conditions (C) and salt stress (S), the relative conductivity, MDA, and Pro concentrations tended to rise with the treatment duration, with notable increases in the relative conductivity, MDA, and Pro contents after the 7th and 14th d of the S treatment as in contrast to the C treatment (*p* < 0.05). However, compared with the S treatment, the S+MT treatment significantly reduced the conductivity (Figure 3G,J) and MDA content (Figure 3H,K) and increased the Pro content in both maize leaves, with the Pro content of ZD958 increasing by 12.71% and 7.14% after the 7th and 14th d of the S+MT treatment (Figure 3I) and that of DMY1 increasing by 10.78% and 11.70% after the 7th and 14th d of the S+MT treatment (Figure 3L). Following 7 d and 14 d of S+MT treatment, the Pro content rose by 10.78% and 11.70%, respectively (Figure 3L).

### 2.4. Melatonin Improves K^+^ Content and K^+^/Na^+^ Ratio

Salt stress severely affects the homeostasis of ions in plants, causing the excessive accumulation of Na^+^ and loss of K^+^ in plant cells, thus causing ion toxicity. To define the effect of melatonin on ionic homeostasis, we measured the Na^+^ and K^+^ content in the maize leaves under each treatment condition. The results showed that the Na^+^ content of the S-treated maize was significantly higher than that of the maize grown under C treatment conditions, and the Na^+^ content gradually increased with the prolongation of the treatment time. Compared with the C treatment, the Na^+^ content of ZD958 was increased by 128.67% and 131.46% after 7 and 14 d of the S treatment, respectively, and the Na^+^ content of DMY1 was increased by 70.66% and 115.29% after 7 and 14 d of the S treatment, respectively. This indicates that ZD958 appeared to respond earlier to salt stress compared to DMY1, and that salt stress had a greater effect on the ionic homeostasis of ZD958. However, the exogenous spraying of melatonin reduced Na^+^ accumulation in the maize leaves under the S treatment and C treatment conditions (Table 1). In addition, the S treatment reduced the K^+^ content in the leaves, whereas exogenous spray melatonin improved the K^+^ content only in the leaves under salt stress (S). Compared to the C treatment, the C+MT treatment exhibited a higher K^+^/Na^+^ ratio in the maize leaves, whereas the S treatment exhibited a lower K^+^/Na^+^ ratio. The K^+^/Na^+^ ratio was significantly increased (*p* < 0.05) in the maize leaves under the S+MT treatment conditions compared to the S treatment. This shows that the exogenous spraying of melatonin attenuates salt stress injury to maize seedlings mainly by maintaining ionic homeostasis and reducing the excess accumulation of Na^+^.

### 2.5. Melatonin Enhances Antioxidant Enzyme Activity

**Figure 4:**
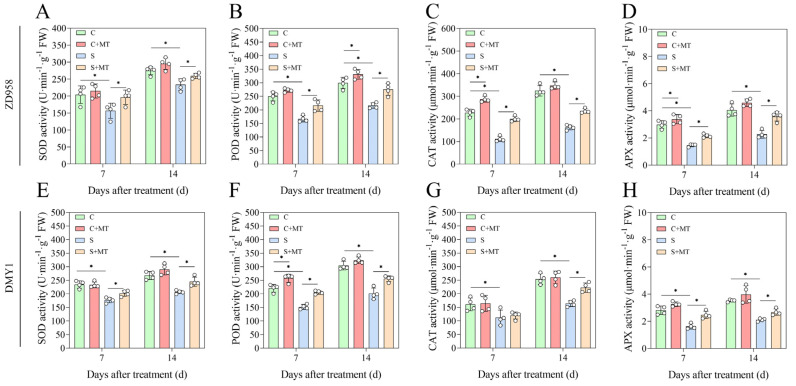
Effects of exogenous melatonin on the activity of antioxidant enzymes in salt-stressed maize seedlings. (**A**,**E**) ZD958 and DMY1 leaves SOD enzyme activity varies after 7 d and 14 d of incubation, respectively; (**B**,**F**) ZD958 and DMY1 leaves POD enzyme activity varies after 7 d and 14 d of incubation, respectively; (**C**,**G**) ZD958 and DMY1 leaves CAT enzyme activity varies after 7 d and 14 d of incubation, respectively; and (**D**,**H**) ZD958 and DMY1 leaves APX enzyme activity varies after 7 d and 14 d of incubation, respectively. Asterisks (*) denote significant differences between treatments, and the data are presented as means ± SE (n = 4) (* *p* < 0.05).

One way that plants react to abiotic oxidative stress under salt stress is by altering the activities of these antioxidant enzymes. Two types of maize were treated differently in this study to measure the activities of several antioxidant enzymes (SOD, POD, CAT, and APX) [41]. The results showed that the activity of the antioxidant enzymes increased gradually with the increase in the treatment time. Compared to the C treatment, the C+MT treatment resulted in a slight increase in antioxidant enzyme activity. The activities of these antioxidant enzymes were lower under the S treatment conditions (Figure 4), where the SOD, POD, CAT, and APX activities in the ZD958 maize leaves were reduced by 17.67%, 27.86%, 49.18%, and 44.01% after 14 d and in the DMY1 maize leaves by 23.52%, 33.91%, 35.48%, and 41.95%, respectively. However, the S+MT treatment resulted in a significant increase in antioxidant enzyme activities (*p* < 0.05). Compared to the S treatment, the activities of SOD, POD, CAT, and APX increased by 13.56%, 27.27%, 42.74%, and 58.27%, respectively, in the ZD958 maize leaves and by 17.36%, 27.22%, 35.95%, and 28.19%, respectively, in the DMY1 maize leaves after 14 d of the S+MT treatment. This shows that melatonin had a greater effect on increasing the antioxidant enzyme activity of ZD958, and also suggests that melatonin can enhance salt tolerance in maize by increasing the antioxidant enzyme activity.

### 2.6. Melatonin Induces the Expression of Antioxidant Enzyme-Related Genes

**Figure 5:**
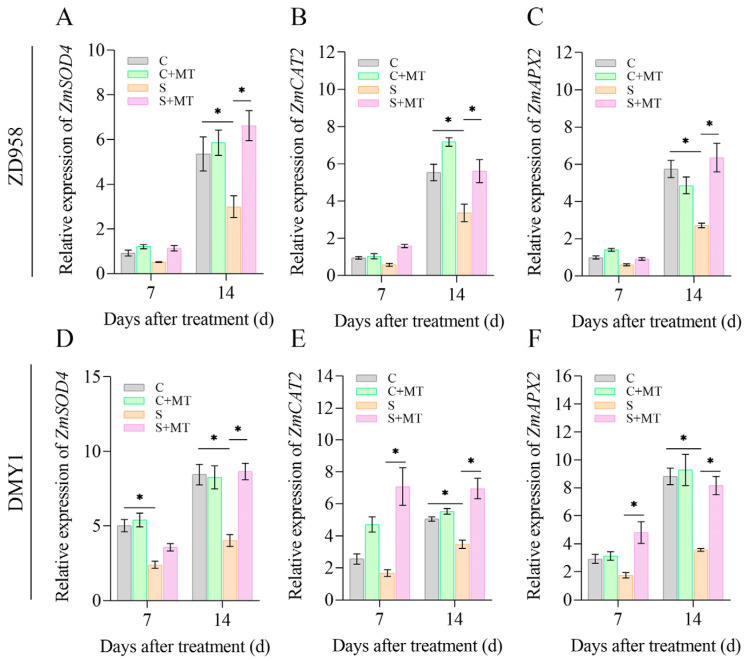
*ZmSOD4*, *ZmCAT2*, and *ZmAPX2* relative expression levels in maize leaves following 7 d and 14 d of melatonin spraying under control and salt stress. (**A**,**D**) Expression levels of *ZmSOD4* in maize leaves; (**B**,**E**) expression levels of *ZmCAT2* in maize leaves; and (**C**,**F**) expression levels of *ZmAPX2* in maize leaves. Asterisks (*) denote significant differences between treatments, and the information is displayed as means ± SE (n = 3). (* *p* < 0.05).

To further explore the regulatory mechanism of melatonin in response to salt stress in maize, we measured the relative expression levels of the antioxidant enzyme-related genes *ZmSOD4*, *ZmCAT2*, and *ZmAPX2* in parallel (Figure 5). With an increase in the incubation time, the S treatment reduced the expression levels of *ZmSOD4*, *ZmCAT2*, and *ZmAPX2* in the ZD958 and DMY1 seedlings compared with the C treatment, in which the expression of *ZmSOD4* was reduced by about 2-fold at 7 d in DMY1 (Figure 5D). Compared with the S treatment, the S+MT treatment increased the expression levels of *ZmSOD4*, *ZmCAT2*, and *ZmAPX2*, with an approximately 3.8-fold increase in the expression of *ZmCAT2* at 7 d in DMY1 and only an approximately 1.8-fold increase in ZD958 (Figure 5B,D). Although the expression level of *ZmCAT2* at 14 d in DMY1 was close to that at 7 d, the expression level of *ZmCAT2* at 14 d in ZD958 was increased by approximately 2.6-fold compared to 7 d (Figure 5B). In addition, the expressions of *ZmSOD4* and *ZmAPX2* were maintained at high levels in both varieties after 14 d of the S+MT treatment compared with the S treatment.

### 2.7. Analysis of Correlation Between Different Measured Parameter of Two Varieties

**Figure 6:**
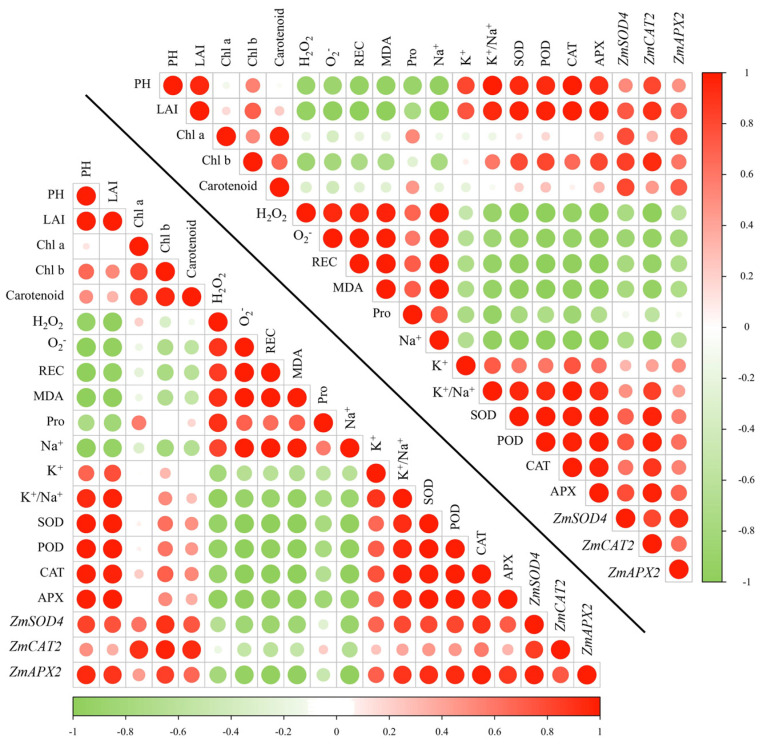
Correlation between different measures in two maize seedlings (ZD958 in the lower left and DMY1 in the right) under salt stress (100 mM NaCl).

Correlation analysis among different maize traits showed strong correlations between traits in the maize leaves. In both maize varieties, oxidative stress indicators (H_2_O_2_ and O_2_^−^) and REC, MDA, and Na^+^ concentrations were negatively correlated with phenotypic traits, K^+^ concentrations, K^+^/Na^+^ ratios, physiological indicators, and the expression of antioxidant enzyme-related genes (Figure 6).

## 3. Discussion

Modern agricultural producers mainly employ direct planting techniques for maize crops, and since seed germination and seedling growth depend, to a large extent, on field conditions after sowing, especially salt-dominated saline, low temperature, drought, and other adversity conditions, maize seed germination, seedling growth, and yield formation will be seriously and adversely affected [42]. Higher plants have evolved many methods and techniques to withstand adverse stress. Under salt stress, melatonin, a multipurpose phytohormone, has been demonstrated to have significant regulatory effects on plant development, as well as physiological and biochemical characteristics [43]. Hussain et al. used melatonin priming to treat saline plant seeds *Zygophyllum simplex* (Zygophyllaceae) and *Portulaca oleracea* (Portulacaceae) and found that melatonin priming increased the rate of seed germination in salt stress and promoted seedling growth in saline plants [44]. Yan et al. discovered that exogenous spraying with melatonin could improve salt tolerance by regulating the expression of genes in photosynthetic capacity, photoprotection, and antioxidant-enzyme-related pathways in wheat [37]. However, little is known about how combined treatments via both melatonin priming and exogenous spraying affect maize seed germination and seedling development under salt stress. Thus, using melatonin-primed maize seeds and foliar spray combination treatments, we carefully examined the relationship between salt tolerance and melatonin-mediated ROS homeostasis and antioxidant capacity.

Salt stress, which is a major form of abiotic stress, significantly inhibits normal plant growth by reducing plant biomass, root growth, and photosynthetic capacity, ultimately leading to yield loss [45]. Melatonin has been described to relieve the adverse impacts of salt stress by increasing plant height, stem thickness, leaves, and biomass accumulation in the root system of plants [46]. The radicle is the first tissue to encounter and sense adversity stress, and the reshaping of root architecture under adversity stress is crucial for plant tolerance [47]. It has been shown that exogenous melatonin increased the root length in Brassica napus [48], maize [38], wheat [37], and alfalfa [49] under salt stress and positively affected vegetative growth in pea (*Pisum sativum* L.) [50] and watermelon (*Citrullus lanatus* L.) [51] by alleviating the impact salt stress on seedling growth traits inhibitory effect on the growth characteristics of seedlings and effectively improving seedling acclimatization to salt stress. In this study, different concentrations of salt stress reduced the germination rate of the test varieties ZD958 and DMY1 at the seed germination stage and affected ZD958 more severely, but melatonin improved the germination rate of the two varieties under salt stress while promoting the elongation and growth of radicles, thus increasing the accumulation of radicle biomass (fresh and dry weights) (Figure 1), which was similar to the results of the previous study.

As two different types of photosynthetic pigments in plants, chlorophyll and carotenoids play key roles in the absorption and transport of light energy [52]. According to reports, some chlorophylls have the ability to convert light energy into chemical energy at light reaction centers, while other chlorophylls and carotenoids attach to light-harvesting complexes (LHCs) in order to absorb and transmit light energy [53,54,55]. Carotenoids may act as photoprotectors, releasing excess energy before plant cells are destroyed [56]. One of the most crucial processes in plants’ reactions to salt stress is thought to be ion homeostasis [57]. Na^+^ affects ion distribution during seedling growth and adversely affects the ionic homeostasis of the plant, especially as excess Na^+^ leads to the degradation of photosynthetic pigments [58,59], and salt stress increases the Na^+^ content and decreases the K^+^ content and K^+^/Na^+^ ratio in plants. It has been shown that melatonin treatment increases carotenoid and chlorophyll contents and enhances photosynthesis in cucumber seedlings [60] and alfalfa [49] under salt stress conditions, in addition to increasing the K^+^ content and K^+^/Na^+^ ratio in begonia and oilseed rape [17,61,62,63], which improves the salt tolerance of plants. In this study, salt stress inhibited the carotenoid and chlorophyll contents and biomass accumulation in the two maize seedlings, with a continuous increase in the Na^+^ content and a continuous decrease in the K^+^ content, especially during the early growth of seedlings, where the Na^+^ content in ZD958 was higher than that in DMY1, which may have been due to the fact that ZD958 appeared to respond to salt stress earlier, resulting in a greater effect of salt stress on the ionic homeostasis of ZD958 caused by the higher Na^+^ content in ZD958 than in DMY1. However, the combination of melatonin-primed and exogenous spray treatments significantly increased the carotenoid and chlorophyll contents, increased the leaf area index (Figure 2), decreased the Na^+^ content, increased the K^+^ content and K^+^/Na^+^ ratio, and promoted the growth of the maize seedlings under salt stress, which may be attributed to the disruption of the fine structure of chloroplasts due to the accumulation of excess Na^+^ resulting from salt stress, which can be alleviated by melatonin. This is consistent with previous findings, where Li et al. used melatonin treatment to alleviate the inhibitory effect of salt stress on leaf photosynthesis, as well as biomass accumulation [17], suggesting that melatonin contributes to the opening of the reopened stomata and improves stomatal function under salt stress.

NaCl-induced oxidative stress is a key factor in cell membrane damage, lipid peroxidation, electrolyte leakage, and imbalances in essential nutrient uptake [64]. In previous studies, salt stress was found to significantly increase the ROS and MDA content in alfalfa; treatment with melatonin caused a significant decrease in MDA content and membrane permeability in salt-stressed banana seedlings [65]. According to reports, melatonin prevents oxidative damage to cell membranes and lowers the generation and buildup of ROS in salt stress situations [66]. In this study, salt stress increased the MDA content, ROS (H_2_O_2_ and O_2_^−^) content, and Pro content, whereas the combination of melatonin seed initiation and exogenous spray treatments reduced MDA and ROS accumulation in both maize species under salt stress (Figure 3), suggesting that the melatonin-treated maize seedlings had a lower cell death, which may contribute to the improvement of antioxidant enzyme activities. In addition, by Pearson correlation analysis, the MDA content, ROS (H_2_O_2_ and O_2_^−^) content, Pro content, and Na^+^ concentration were negatively correlated with other indicators (Figure 6), which is similar to the results of previous studies [49,65].

**Figure 7:**
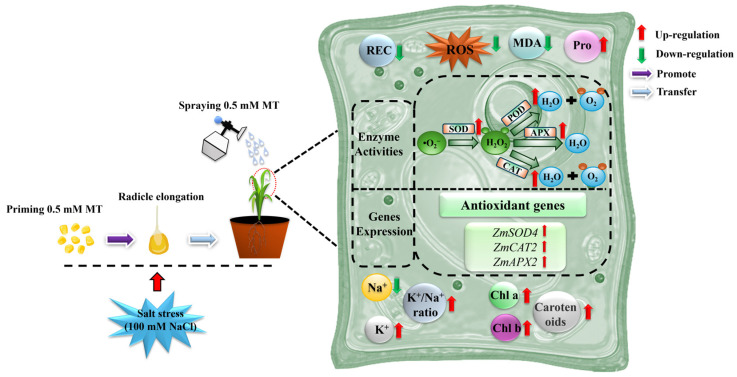
Modeling the effect of combined melatonin seed priming and exogenous spray treatments on maize seedling growth stressed by salt.

Salt stress destroys the ROS balance in plants, leading to too little or too much ROS accumulation and a series of oxidative damage, thus affecting the normal life activities of plants. Plants have evolved effective antioxidant defense systems to reduce the accumulation of reactive oxygen species (ROS) and oxidative damage brought on by abiotic stress. These mechanisms include an increased activity of antioxidant enzymes like SOD, POD, CAT, and APX, where SOD converts O_2_^−^ into H_2_O_2_, which POD then scavenges in the extracellular compartment, CAT converts H_2_O_2_ into H_2_O and O_2_, and APX uses ascorbic acid as a donor to remove H_2_O_2_ [34,67]. To verify further the effect of melatonin on antioxidant capacity during the growth of the maize seedlings, we investigated the changes in the activity of antioxidant enzymes associated with ROS scavenging and the expression of the antioxidant enzyme genes *ZmSOD4*, *ZmCAT2*, and *ZmAPX2*. It has been demonstrated that *ZmSOD4*, *ZmCAT2*, and *ZmAPX2* contribute to maize’s reaction to abiotic stressors [68]. We found that salt stress reduced the activities of the SOD, POD, CAT, and APX antioxidant enzymes in both varieties (Figure 4), and that salt stress reduced the expressions of *ZmSOD4*, *ZmCAT2*, and *ZmAPX2*. However, when treated with a combination of melatonin-primed and exogenous sprays, the expressions of *ZmSOD4*, *ZmCAT2*, and *ZmAPX2* were all significantly increased (Figure 5), especially during the early stage of seedling treatment (7 d). The expression of *ZmCAT2* in the DMY1 variety was rapidly elevated, however, with the prolongation of the treatment time, the expression remained almost unchanged, but ZD958 showed a rapid elevation of this trend. However, further exploration is still needed in the future regarding how melatonin specifically affects *ZmCAT2* expression in both varieties. Combined with the ROS and MDA contents, this shows that melatonin scavenges excessive ROS accumulation due to salt stress by inducing antioxidant enzyme activities to maintain cell membrane stability and reduce cell membrane damage, while saving the energy required by maize seedlings to resist salt stress to initiate other growth and metabolic activities. Similar to the current findings of this study, it has been demonstrated that exogenous melatonin increases the activity of antioxidant enzymes under abiotic stress [17,66]. Melatonin use also increased the activities of the SOD, POD, CAT, and APX enzymes in cucumber seedlings (*Cucumis sativus* L.) [69] and tomato (*Solanum lycopersicum* L.) [70] under salt stress. Since melatonin seed initiation and exogenous spraying can increase SOD, POD, CAT, and APX activities, as well as the expression of genes encoding antioxidant enzyme-related genes to improve the salt tolerance of maize seedlings under salt stress, we assume that elevated SOD, POD, CAT, and APX activities are associated with an increased salt tolerance. Thus, melatonin seed priming and exogenous spraying together may help to reduce the damage caused by salt stress to the ROS scavenging system of maize seedlings and increase their antioxidant capacity, which could be a key strategy for enhancing their salt tolerance (Figure 7).

## 4. Materials and Methods

### 4.1. Plant Material and Chemicals

All experiments were conducted at Heilongjiang Bayi Agricultural University (31°24 N, 121°29 E); the maize varieties tested were Zhengdan 958 (ZD958, dent maize) and DMY1 (DMY1, flint maize), which were provided by the Maize Germplasm Innovation Research Laboratory of Heilongjiang Bayi Agricultural University. All seeds were harvested in November 2023 and subsequently stored in a seed storage cabinet (4 °C), where seed viability was preserved. Melatonin, Nitrotetrazolium Blue chloride (NBT), and 3,3-diaminobenzidine (DAB) were bought from Sigma-Aldrich (St. Louis, MO, USA). All sterile water used in this study was distilled water sterilized at a high temperature (120 °C).

### 4.2. Seed Germination Test

We sterilized the maize seeds according to our previous methods [34]. In order to screen for appropriate salt concentrations, the maize seeds were separately placed in paper-bed germination boxes (L × W × H: 14 cm × 14 cm × 6 cm) that contained sterile water (10 mL) (control) or concentrations of salt (50, 100, 150, and 200 mM NaCl), and incubated in an artificial climate chamber (DRX-1000, Ningbo Dongnan Instrument Co., Ltd., Ningbo, China) in the dark (temperature 25 ± 0.5 °C, relative humidity 50 ± 5% RH). Over the course of 72 h, dynamic changes in seed germination (radicle penetration of the seed coat) with varying salt concentrations were noted every 12 h.

In order to analyze how melatonin affects salt stress, the maize seeds were immersed in melatonin solution at varying concentrations (0, 0.1, 0.5, and 1.0 mM) for 24 h (in the dark) [71]. Following room-temperature drying, 25 triggered seeds were put in paper-bed germination cassettes with either 100 mM NaCl solution or 10 mL of sterile water, and the samples were incubated in the dark (temperature 25 ± 0.5 °C, relative humidity 50 ± 5% RH) for 72 h in an artificial incubator (DRX-1000, Ningbo Dongnan Instrument Co., Ltd., Ningbo, China). Using sterile water-initiated maize seeds as a control, the germination rate, root length, root fresh weight, and dry weight of the maize seeds were measured. Four duplicates of each treatment were made, with 25 seeds inside every germination box serving as one replication.

### 4.3. Salt Treatment and Melatonin Application in Maize

**Figure 8:**
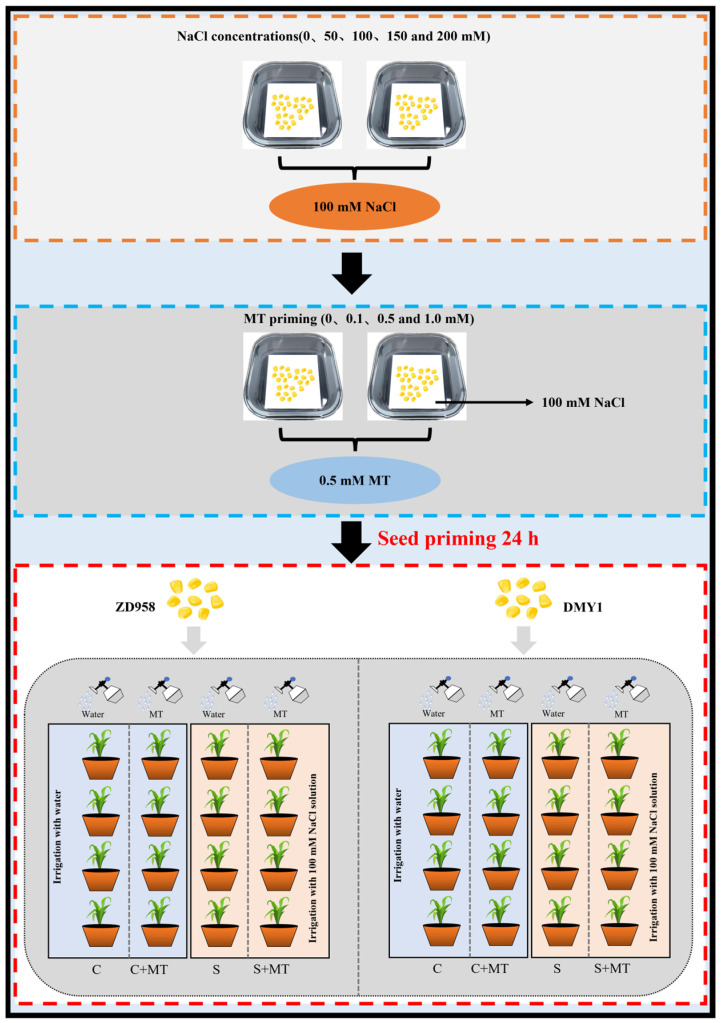
Schematic of the study design.

Maize seeds with a homogeneous seed size were selected and placed in 0.5 mM melatonin solution to be triggered under dark conditions for 24 h. After being dried at room temperature, the seeds collected were evenly and uniformly sown in plastic pots (L × W × H: 13 cm × 13 cm × 15 cm) containing 2 kg of farm soil (Supplementary Table S1) and maintained under a growth condition in a growth chamber with a logarithmic photoperiod of 16/8 h (light/dark), a light intensity of 200 μmol·m^−2^·s^−2^, and a temperature of 25 ± 2 °C. All plastic pots were divided into the following four treatment groups: C (control), C+MT (control + melatonin), S (salt treatment), and S+MT (salt treatment + melatonin); 50 mL of tap water was used to irrigate the pots for C and C+MT at 1 d intervals, while 50 mL of a 100 mM NaCl solution was used for S and S+MT. The maize leaves were treated with melatonin sprays after 7 d of growth; C+MT and S+MT were sprayed with 50 mL of 0.5 mM melatonin solution every evening via foliar spray; and for 14 d, C and S received foliar spray treatments every evening using 50 mL of tap water (Figure 8).

### 4.4. Determination of Plant Growth Characteristics

The characteristics of plant growth were assessed 7 d and 14 d following melatonin spraying. Fresh samples were separated from potted plants, the Presica LS120A (Sartorius AG, Göttingen, Germany) was used to measure the fresh samples’ fresh weight (FW) and dry weight (DW), plant height (PH) was measured using a straightedge, and measurements were also taken of the greatest leaves’ length and width. With four replications for every treatment, leaf area was computed using the formula leaf area = (leaf length × leaf width × 0.75).

### 4.5. Determination of Relative Electrical Conductivity (REC), Malondialdehyde (MDA), and Proline (Pro) Contents

For the relative electrical conductivity (REC) measurement, the strategies of the study by Lutts et al. were referred to and briefly modified [72]. Shortly after being weighed, a 10 mL centrifuge tube was stuffed with sterile water and 0.1 g of fresh maize leaves. After 24 h, initial conductivity (R1) was determined at room temperature using a conductivity meter (FiveEasy F30, Mettler-Toledo Ltd., Australia). The centrifuge tubes containing the samples were then boiled in a water bath for 30 min and cooled to room temperature to determine the final conductivity (R2), with four replicates for each treatment. Relative electrical conductivity (REC) was calculated using the formula R1/R2 × 100%.

Lipid peroxidation levels were assessed using thiobarbituric acid (TBA) as a reactant to calculate malondialdehyde (MDA) content in leaves based on Heath and Packer’s approach [73].

The standard program outlined by Tiwari et al. [74] was used to measure the amount of proline (Pro) in maize leaf tissues.

### 4.6. Localization and Quantification of H_2_O_2_ and O_2_^−^

The histochemical detection of H_2_O_2_ based on H_2_O_2_-catalysed polymerization of 3,3-diaminobenzidine (3,3-diaminobenzidine, DAB) was performed with reference to the approach outlined by Xia et al. [75]. The H_2_O_2_ content in leaves was realized by a colorimetric method with reference to the approach outlined by Zhang et al. [76].

Following Dunand et al. [77] procedure, the leaves were submerged in a 100 mM nitroblue tetrazolium (NBT) solution mixed in 50 mM phosphate buffer (pH = 7.5) to obtain O_2_^−^ staining. Using the Ke et al. [78] approach, the absorbance value at 520 nm of the dark red azo product produced by the hydroxylamine reaction was used to determine the O_2_^−^ content in the leaves.

### 4.7. Determination of Chlorophyll a, Chlorophyll b and Carotenoid Content

Based on the methodology outlined by Arnon et al. [79], fresh leaves under different treatments were individually cut into pieces and, subsequently, the pieces (0.1 g) were placed in centrifuge tubes containing 95% (*v*/*v*) ethanol solution and allowed to stand under dark conditions until the leaf samples were completely discolored and finally, the Specord Plus 210 (Analytik Jena AG, Jena, Germany) to determine the absorbance values at 665 nm, 649 nm, and 470 nm.

### 4.8. Determination of Na^+^ and K^+^ Content

A flame spectrophotometer (Model 420, Sherwood Scientific, Cambridge, UK) was used to determine the Na^+^ and K^+^ content of the leaves using the Cen et al. method [25]. For every treatment, four replications were conducted.

### 4.9. Assessment of the Activity of Antioxidant Enzymes

To investigate the antioxidant enzyme activity, intact leaves of maize seedlings from various treatments were gathered, quickly submerged in liquid nitrogen, and kept at −80 °C. Gallardo et al.’s approach was used to measure the SOD activity [80], Tan et al.’s approach was used to measure POD [81], the approach outlined by Mhamdi et al. was used to measure CAT activity [82], and APX activity was assessed using Nakano and Asada’s approach [83].

### 4.10. RNA Extraction and qRT-PCR

TRIzol^®^ reagent (Invitrogen, Carlsbad, CA, USA) was used to extract the total RNA from the maize leaves at 7 d and 14 d following the various treatments. The ReverTra AceTM qPCR RT Master Mix with gDNA Remover kit (TOYOBO Co., Osaka, Japan) was used for first-strand cDNA synthesis, ysing the previously reported *ZmActin* [68] as an internal reference gene (Supplementary Table S2). Lastly, using the Bio-Rad CFX96 Real-Time Fluorescence Quantitative PCR Detection System (Bio-Rad, Hercules, CA, USA) and the SYBR^®^ Green PCR Master Mix (TOYOBO Co., Osaka, Japan), real-time fluorescence quantitative PCR (qRT-PCR) studies were performed in accordance with the instructions. Three biological replicates (three technical replicates per sample) were used for each treatment. The 2^−ΔΔCt^ technique was used to calculate relative gene expression [84].

### 4.11. Data Analysis

Software called SPSS 19.0 (SPSS Inc., Chicago, IL, USA) was used to conduct the Pearson correlation analyses and analyses of variance (ANOVAs). Duncan’s new multiple range test was used to examine the significance of differences between treatment groups; asterisks (*), and various letters denote significant differences between treatments. (* *p* < 0.05, ** *p* < 0.01).

## 5. Conclusions

In conclusion, the combination of melatonin seed priming and exogenous spraying treatment (0.5 mM) can effectively alleviate the reduction in the seed germination rate and seedling growth delay of the test varieties ZD958 and DMY1 induced by salt stress (100 mM NaCl), reduce the overaccumulation of salt stress-induced ROS, increase the content of photosynthetic pigments, K^+^ content, and the K^+^/Na^+^ ratio, and alleviate the oxidative damage induced by salt stress. These results indicate that salt stress-induced oxidative damage was reduced. In this study, we found that exogenous melatonin increased the activities of the antioxidant enzymes SOD, POD, CAT, and APX, as well as the expressions of related genes *ZmSOD4*, *ZmAPX2*, and *ZmCAT2*, to improve the salt tolerance in maize seedlings under salt stress. This provides a new idea to study how melatonin, a novel bioactive molecule, can improve the technical innovation of maize seed germination ability and salt tolerance.

## Figures and Tables

**Table 1:** Differential analysis of the K^+^, Na^+^, and K^+^/Na^+^ ratios in maize leaves under various conditions.

Variety	Days	Treatment	Na^+^ Content (mg·g^−1^)	K^+^ Content (mg·g^−1^)	K^+^/Na^+^ Ratio
ZD958	7	C	18.72 ± 0.33 c	18.24 ± 1.03 a	0.97 ± 0.04 a
		C+MT	13.59 ± 0.53 d	12.91 ± 0.84 b	0.95 ± 0.04 a
		S	42.81 ± 0.76 a	9.29 ± 1.03 c	0.22 ± 0.03 c
		S+MT	32.63 ± 0.84 b	12.26 ± 0.99 b	0.38 ± 0.01 b
	14	C	38.05 ± 0.73 c	58.01 ± 2.52 a	1.52 ± 0.02 b
		C+MT	25.97 ± 0.63 d	46.05 ± 3.22 b	1.77 ± 0.02 a
		S	88.07 ± 0.64 a	38.01 ± 0.87 d	0.43 ± 0.01 d
		S+MT	56.89 ± 1.33 b	41.49 ± 1.99 c	0.73 ± 0.01 c
DMY1	7	C	17.38 ± 0.93 c	16.24 ± 1.32 a	0.93 ± 0.05 a
		C+MT	13.36 ± 0.73 d	10.91 ± 1.28 b	0.82 ± 0.05 b
		S	29.66 ± 0.88 a	7.28 ± 0.32 c	0.25 ± 0.04 d
		S+MT	19.23 ± 0.36 b	10.26 ± 0.76 b	0.53 ± 0.03 c
	14	C	24.66 ± 1.42 c	37.98 ± 2.65 a	1.54 ± 0.03 a
		C+MT	17.38 ± 0.33 d	36.02 ± 3.21 a	2.07 ± 0.04 b
		S	53.09 ± 2.32 a	19.27 ± 2.11 c	0.36 ± 0.02 d
		S+MT	37.80 ± 1.32 b	31.62 ± 2.98 b	0.84 ± 0.02 c

Different lowercase letters indicate significant differences at the *p* ≤ 0.05 level.

## Data Availability

Data are contained within the article and Supplementary Materials.

## Supplementary Materials

The following supporting information can be downloaded at: https://www.mdpi.com/article/10.3390/plants14020296/s1, Table S1: Physico-chemical properties of the agricultural soils used in the experiment; Table S2: Specific primer sequences for qRT-PCR.
