# Supplementary material for: Melatonin Enhances Maize Germination, Growth, and Salt Tolerance by Regulating Reactive Oxygen Species Accumulation and Antioxidant Systems
Source: Plants (Basel). 2025 Jan 20;14(2):296. doi: 10.3390/plants14020296 (PMC11768311; doi:10.3390/plants14020296)
Supplement: Supplementary file 1 [file plants-14-00296-s001.zip › plants-3371825-supplementary.pdf]

Melatonin Enhances Maize Germination, Growth, and Salt Tolerance by Regulating Reactive Oxygen Species Accumulation and Antioxidant Systems

Wei-Qing Li [1,†], Jia-Yu Li [1,†], Shao-Jie Bi [2,3], Jia-Yue Jin [2], Zhong-Ling Fan [2], Zi-Lin Shang [2], Yi-Fei Zhang [1], Yan-Jie Wang [1,2,3*]

Supplementary Table S1 Physico-chemical properties of the agricultural soils used in the experiment

Classification	Activity
Total nitrogen ($g{\cdot}kg^{-1}$)	1.19
Alkali hydrolyzed nitrogen ($mg{\cdot}kg^{-1}$)	93.05
Organic matter ($g{\cdot}kg^{-1}$)	9.28
Available Phosphorus ($mg{\cdot}kg^{-1}$)	25.44
pH	7.18
Fast-acting potassium ($mg{\cdot}kg^{-1}$)	173.00

Note: The value is the mean ± standard deviation of 4 repetitions.

Supplementary Table S2 Specific primer sequences for qRT-PCR

Primer name	Primer sequence (5'-3')
ZmSOD4-F	GATCTTGGAAAGGGTGGACA
ZmSOD4-R	GAAGTCCAGCGACCCATTT
ZmAPX2-F	CCCATCCTATCCTACGCTGA
ZmAPX2-R	ATCAGGTCCGCCGGTTAC
ZmCAT2-F	ACGACATCACCCACCTGAC
ZmCAT2-R	GGAGAAGCGGACGATGAC
ZmActin-F	CGTTACCGGCTCATTG
ZmActin-R	AGGGAGCACCAGACTCAT
